# Safety and efficacy of autologous, adipose-derived mesenchymal stem cells in patients with rheumatoid arthritis: a phase I/IIa, open-label, non-randomized pilot trial

**DOI:** 10.1186/s13287-022-02763-w

**Published:** 2022-03-03

**Authors:** Ridhima Vij, Kevin A. Stebbings, Hosu Kim, Hyeonggeun Park, Donna Chang

**Affiliations:** 1 Hope Biosciences Stem Cell Research Foundation, 16700 Creek bend Dr., Sugar Land, TX 77478 USA; 2 Hope Biosciences, Sugar Land, TX USA

**Keywords:** Adipose-derived mesenchymal stem cell, Autologous, Clinical trial, Rheumatoid arthritis, Intravenous, Efficacy

## Abstract

**Objective:**

The present study is a phase I/IIa non-randomized, open-label study to evaluate safety and efficacy of a single, intravenous infusion of autologous, adipose-derived mesenchymal stem cells (adMSCs) over a period of 52 weeks, in patients with active rheumatoid arthritis (RA).

**Methods:**

15 eligible RA patients aged 18–65 years were enrolled and followed up at weeks 4, 12, 26 and 52 after receiving a single intravenous dose of 2 × 10^8^ adMSCs. Efficacy was examined using American College of Rheumatology (ACR66/68 score) criteria for swollen and tender joint counts (S/TJC), and serum TNF-α, IL-6, CRP, and ESR levels. Safety endpoints included measures of hematologic, hepatic, and renal function.

**Results:**

ACR66/68 scores for both S/TJC showed significant improvements with large effect sizes (ES) at week 52 vs baseline (*p* < 0.01, ES = 0.83 and *p* < 0.001, ES = 0.93 respectively). Medium to large ES were also obvious for ACR66/68 scores measured at other timepoints. Levels of inflammatory markers, TNF-α, IL-6 and ESR remained unchanged compared to baseline. However, a difference in CRP levels with a small effect size was observed at week 4 (*p* = 0.229, ES = 0.33) with further improvement at week 52 (*p* = 0.183, ES = 0.37). Post-intervention, levels of hematologic, hepatic, and renal function remained largely unchanged (*p* > 0.05). No acute or long-term serious adverse events (AEs) occurred.

**Conclusions:**

The results indicated that a single, intravenous administration of autologous adMSCs is safe and efficacious for improvement in joint function in patients with active RA. Data from the current study supports the exploration of ad-MSCs as a therapeutic intervention for RA.

*Trial Registration* Clinical trial registration number: NCT03691909. Registered September 27, 2018- Retrospectively registered (https://clinicaltrials.gov/show/NCT03691909).

**Supplementary Information:**

The online version contains supplementary material available at 10.1186/s13287-022-02763-w.

## Introduction

Rheumatoid arthritis (RA) is a chronic, inflammatory autoimmune disease associated with joint pathogenesis, bone and cartilage deformities as well as systemic comorbidities [1], affecting about 1% of the population worldwide [2]. Some of the common symptoms of RA include pain, stiffness, and swelling which are often followed by progressive disability and joint dysfunction [3]. Currently, there is no cure for RA. Successful therapies often begin with corticosteroids, which shutdown the disease process while managing symptoms, until disease-modifying antirheumatic drugs (DMARDs) such as methotrexate, begin to take effect. Patients without a satisfactory response to non-biological DMARDs, either switch or supplement with other synthetic DMARDs and/or one of several increasingly available biological DMARDs, including TNF-α inhibitors, Anti-B/T cell and IL-6R therapies [4–6]. Although not common, these therapies can have serious side effects, including infections, hematologic, hepatic, and renal dysfunction, and bone marrow suppression [7, 8]. Additionally, long-term usage of DMARDs may render the patients resistant, resulting in inefficient therapeutic outcomes.

Mesenchymal stem cells (MSCs) are multipotent progenitor cells which can differentiate into a variety of tissues, including, cartilage, muscle, tendon/ligament, and bone [9–11]. Their ability to modulate immune responses and promote regeneration contribute to the therapeutic impact observed in numerous preclinical inflammatory studies [3, 12–17]. More specifically, MSCs have been implicated to have therapeutic potential with large effect sizes in preclinical models of RA [18]. The use of MSC therapy in RA in clinical trials began more than a decade ago [19], and despite variations in tissue source among the studies, significant improvements in RA severity have been reported [20–23]. Adipose-derived MSCs (adMSCs) have comparable immunomodulatory properties to the MSCs derived from other sources, but are far more practical, cost effective and easy to obtain [24]. However, historically, cell therapies are known to produce varying efficacy results, largely due to donor-to-donor variability and cryopreservation of the final product [25]. The current study employed fresh, undifferentiated, culture-expanded MSCs to ensure a standardized and consistent final product, that could be measured for efficacy in RA. Although therapeutic benefit of autologous ad-MSCs in RA patients has been previously reported [26], this is the first study to illustrate clinically significant reduction in joint dysfunction, demonstrating safety and efficacy of autologous adMSC therapy in the patients with RA.

## Methods

### Trial design and participants

The current trial is a phase I/IIa open-label, non-randomized, pilot trial to test the safety and efficacy of Hope Biosciences’ autologous, adipose-derived mesenchymal stem cells (HB-adMSCs) in RA. The study took place from September 2018 to September 2020, in Houston, Texas. The targeted treatment population were those who had persistent active RA symptoms despite being on a stabilized treatment. Twenty-three (23) subjects were recruited and screened as patients of the clinic or contacted the site through clinicaltrials.gov (https://clinicaltrials.gov/show/NCT03691909). Fifteen (15) patients participated in the study across two (2) clinics in Houston, Texas USA. The trial sponsor covered all costs of stem cell harvesting, production, and trial participation. The study was approved by the Western Institutional Review Board located in Olympia, Washington, and conducted in accordance with Good Clinical Practice guidelines and the Declaration of Helsinki. All participants provided written informed consent.

### Patient eligibility

*Inclusion criteria* (1) males and females; (2) aged 18–65 years; (3) active RA with ≥ 6 swollen AND ≥ 6 tender joints on the ACR66/68 joint assessment; (4) CRP levels > 4.9 mg/L OR ESR > 10 mm/h for men, > 20 mm/h for women; (5) without current established treatment or on a stable regimen for > 4 weeks pre-screening. Patients were required to stay on a stable drug regime throughout the study.

*Exclusion criteria* (1) inability to understand and provide signed informed consent; (2) pregnancy, lactation, or, if female of childbearing potential, positive serum β-hCG at screening; (3) currently diagnosed malignant neoplasm (excluding resolved cancer for ≥ 5 years); (4) uncontrolled systemic illness, including, but not limited to, hypertension (systolic > 150 mm Hg or diastolic > 95 mm Hg), diabetes, renal, hepatic, or cardiac failure or any laboratory abnormality that poses a safety risk; (5) hemoglobin ≤ 8.5 g/dL; (6) white blood cells ≤ 3500/mm^3^ (3.5 G/L); (7) any other illness which, in the opinion of the investigator, characterizes the subject as not being a good candidate for the study; (8) participation in an investigational drug or device trial within 4 weeks prior to treatment or 5 half-lives of the investigational product (whichever is longer); (9) Hepatitis B or C infection, and/or human immunodeficiency virus infection at screening; (10) history of Treponema pallidum infection.

### Autologous HB-adMSC production

adMSCs were purified from 3-5 mL of adipose tissue for each subject. Tissue was treated with collagenase to separate the stromal vascular fraction (SVF). Cells from the SVF were plated in Hope Biosciences’ (HB)-103 medium to establish a P0 culture. The resulting adherent cells were cultured with HB-101. Cells were cryopreserved at passages 0, 1 and 2. For infusions, passage 2 cells were thawed and cultured to passage 4 (Additional file 1: Fig. S1). 2 × 10^8^ HB-adMSCs were freshly harvested from passage 4 cultures and packaged in 20 mL 0.9% sterile saline for administration. Each lot passed cGMP compliant quality control standard assessments and was administered within 48 h of packaging (Additional file 2: Table S1). Quality assessments included viability; appearance; sterility (USP71); gram staining; mycoplasma; endotoxin; and cell identity/purity as indicated by MSC defining surface markers (CD73 + , CD29 + , CD31- and CD45-).

### adMSC administration

Study participants were given a single intravenous infusion of 2 × 10^8^ live cells of HB-adMSCs. 2 × 10^8^ HB-adMSCs was mixed into a 250 mL bag of 0.9% sterile saline solution and then infused through IV administration set and catheter at 83.3 gtts/min for an hour. Clinical and safety parameters were monitored on-site for 4 h post-infusion, after 24 h, and at weeks 1, 4, 8, 12, 26, and 52 following infusion.

### Study endpoints

Primary endpoint assessments for safety included blood panels for hematologic (i.e., CBC, CMP), renal (creatine, BUN), and hepatic function (AST, ALT) performed at baseline, weeks 4, 12, 26, and 52 post-infusion. Secondary endpoints included the assessments of values from ACR 66/68-swollen/tender joint count and inflammatory cytokines (tumor necrosis factor alpha (TNF-α), interleukin-6 (IL-6), C-reactive protein (CRP), and erythrocyte sedimentation rate (ESR), measured at baseline, weeks 4, 12, 26 and 52. Joint assessments were performed by the same investigator for each subject. All laboratory tests were performed by LabCorp, USA.

### Statistical analysis

All the results were analyzed using GraphPad Prism version 9.2.0 for Mac, GraphPad Software (San Diego, California). To determine the normality of the data, the Shapiro–Wilk *W* test was performed. Data for safety analysis at baseline and at follow-up visits were compared by Mixed-Effect Analysis followed by Dunnett’s correction for multiple comparisons. Statistical significance was determined by two-tailed *p* < 0.05. For the efficacy data, the normality test indicated non-normal distribution. Therefore, non-parametric Wilcoxon matched-pairs signed rank test was used to compare the efficacy data at the post-treatment follow-up visits vs baseline. For these analyses, statistical significance was assumed at the *p* value < 0.01 (Holm–Šídák correction with *a* = 0.0125 rather than 0.05 was applied to account for multiple comparisons). All variables were subjected to descriptive analysis, represented as median with inter-quartile range (IQR).

Effect sizes were also reported to determine the clinical significance for efficacy data where statistical significance was observed. The effect size (ES) was calculated for the corrected *p* values, using Rosenthal’s formula: ${\text{ES}} = z/\surd N$ where *N* is the number of subjects [27, 28] and interpreted as small (≥ 0.2), medium (≥ 0.4) and large (≥ 0.8).

## Results

### Patient characteristics

**Fig. 1:**
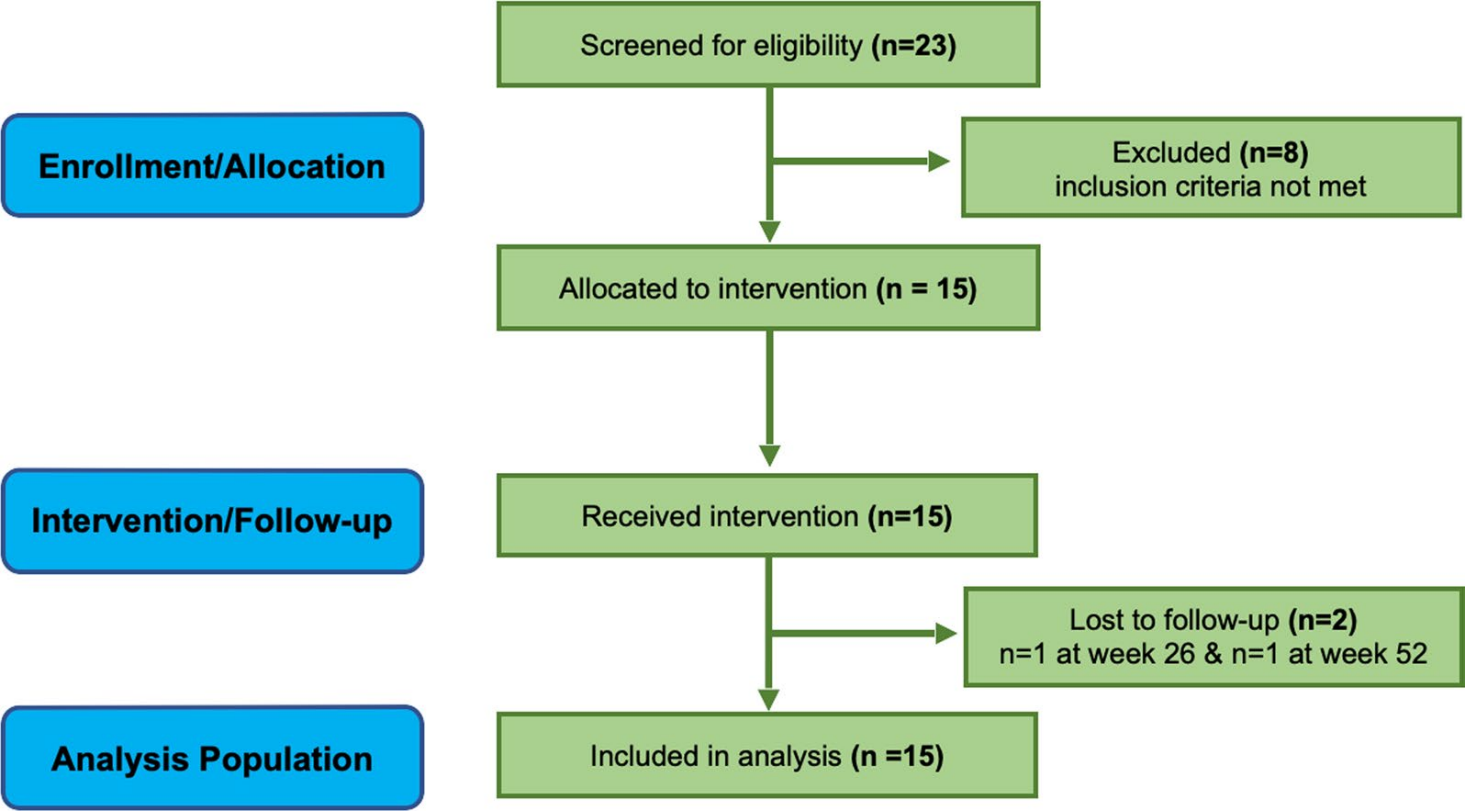
Study flow diagram

Of the 23 patients screened for the study, 8 failed screening (eligibility criteria not met), 15 were enrolled and received treatment. 13 subjects completed all study procedures to study completion at 52 weeks (N = 2 subjects were lost to follow-up one at week 26 and another at week 52) (Fig. 1). The study subjects were predominately female (93.3%), with the median age of 52 years (IQR 38–61). The median duration of illness was 11.4 years with an IQR of 6.20–26.4 (Table 1). Thirteen out of 15 subjects (86.7%) were on DMARDs or Glucocorticoids (12 for ≥ 6 months, 10 for ≥ 1 year, 7 for ≥ 2 years; Additional file 2: Table S2) while sole treatment for 2 of 15 subjects was prophylactic supplements or NSAIDs. Twelve out of 15 subjects had failed at least 1 or more prior DMARD therapies, due to no response or intolerable side effects, with the median of failed therapies being 1.0 (IQR 1.0–3.0); all subjects not failing a therapy had been on DMARD therapy for > 6 months (Additional file 2: Table S2).

**Table 1:** Baseline demographic characteristics of N = 15 subjects

Age (years)	52 (38–61)	
BMI	< 30	6 (40%)
	> 30	9 (60%)
Sex	Female	14 (93.3%)
	Male	1 (6.7%)
Ethnicity	Hispanic or Latino	4 (26.6%)
	Not Hispanic or Latino	11 (73.3%)
Race	Black or African American	1 (6.7%)
	White	14 (93.3%)
Concomitant medications	DMARDs or Glucocorticoids	13 (86.7%)
	Biologic DMARDs	12 (80%)
	Synthetic DMARDs	10 (66.7%)
	Glucocorticoids	6 (40%)
Disease duration (years)		11.4 (6.20–26.4)

BMI, body mass index; DMARDs, disease modifying anti-rheumatic drugs statistics represented: median (IQR); n (%)

### Safety evaluation

#### Safety and tolerability

Lab examination for hematologic measures of all patients showed no significant changes at any of the follow up visits compared to baseline (*p* > 0.05). Comprehensive metabolic panel measures, including total protein, total globulin or albumin also remained unchanged compared to the values prior to intervention. However, there was a minor change observed in albumin to globulin ratio (*p* = 0.047, Additional file 2: Table S3). Overall, renal and liver function remained largely unchanged without any significant changes at any of the follow-up visits compared to baseline. Details of all lab results (descriptive and significance) for safety measures are presented in Additional file 2: Table S3.

#### Adverse events (AEs)

Out of a total of 27 AEs, 15 (55.6%) were classified as mild, 8 (29.6%) as moderate and 4 (14.8%) as severe. Four AEs (all mild): hematuria and right eyelid pruritis (same subject), anemia (1 subject), and thrombocytopenia (1 subject) were classified as treatment related. No immediate post-infusion reactions were observed. 14/15 subjects reported at least 1AE during the study, however some subjects showed disproportionate levels of AEs e.g., one subject accounted for ~ 30% of total events, one subject accounted for 50% of the total severe events while another subject accounted for 50% of the total moderate AEs. No adverse events due to drug interactions were observed.

### Efficacy

#### RA symptoms

**Fig. 2:**
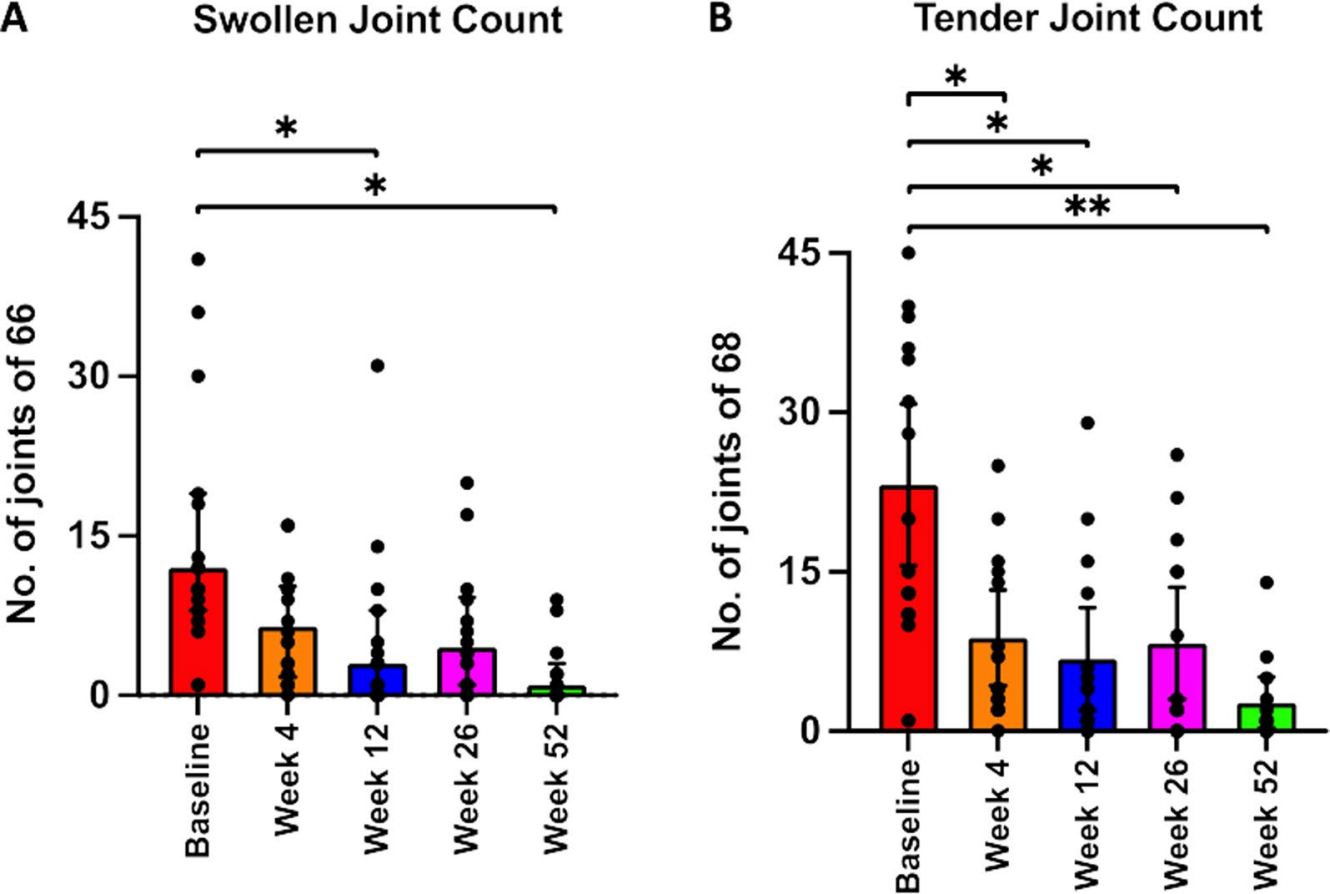
Joint Counts. Swollen joint count showed a significant decrease at each of the follow-up visits compared to baseline. Tender joint scores showed highly significant decline at all follow-up weeks, compared to baseline. Significance defined at *p* value ≤ 0.01 (Holm–Šídák correction for multiple comparisons), *p value < 0.01; **p value < 0.001; Wilcoxon-signed rank test

Both swollen and tender joint scores (measured on ACR 66/68 joint assessment) showed clinically remarkable improvements at the end of follow-up at week 52 vs baseline, with the medians declining from 12.0 (IQR 8.0–19.0) to 1 (IQR 0.0–3.0) for swollen joints and from 20.0 (IQR 11.0–36.0) to 1.0 (IQR 0.0–4.0) for tender joint counts. Efficacy data analyses revealed that the improvements in the joint counts were statistically significant (*p* = 0.003 and *p* = 0.0008) with large effect size (ES = 0.83 and ES = 0.93) for swollen and tender joint counts respectively; Fig. 2, Table 2).

**Table 2:** Median, inter-quartile range and efficacy measures for joint counts and inflammatory parameters at baseline and week 52 post-intervention

Variable	Baseline Median (IQR)	Week 52 Median (IQR)	*p* value	Effect Size
Tender JC	20.0 (11.0–36.0)	1.00 (0.00–4.00)	0.0008^**^	0.93
Swollen JC	12.0 (8.00–19.0)	1.00 (0.00–3.00)	0.003^*^	0.83
CRP	10.0 (4.50–18.1)	6.00 (3.00–12.0)	0.183	0.37
ESR	43.0 (33.0–55.0)	34.5 (23.8–62.8)	0.775	0.05
IL-6	4.90 (2.90–12.1)	4.60 (2.75–13.9)	0.714	0.10
TNF-α	1.45 (0.88–3.23)	1.15 (0.73–2.28)	0.743	0.09

JC, joint count; TNF-α, tumor necrosis factor-alpha; IL-6, interleukin-6; ESR, erythrocyte sedimentation rate; CRP, C-reactive protein; IQR, interquartile range (25%—75%)

^*^p < 0.01 and **p < 0.001 (Wilcoxon-signed rank test; Holm-Šídák correction for multiple comparisons). ES = Effect Size (Rosenthal’s formula with N = 13; one subject had data out to week 26 and one had data out to week 12)

#### Inflammatory measures

**Fig. 3:**
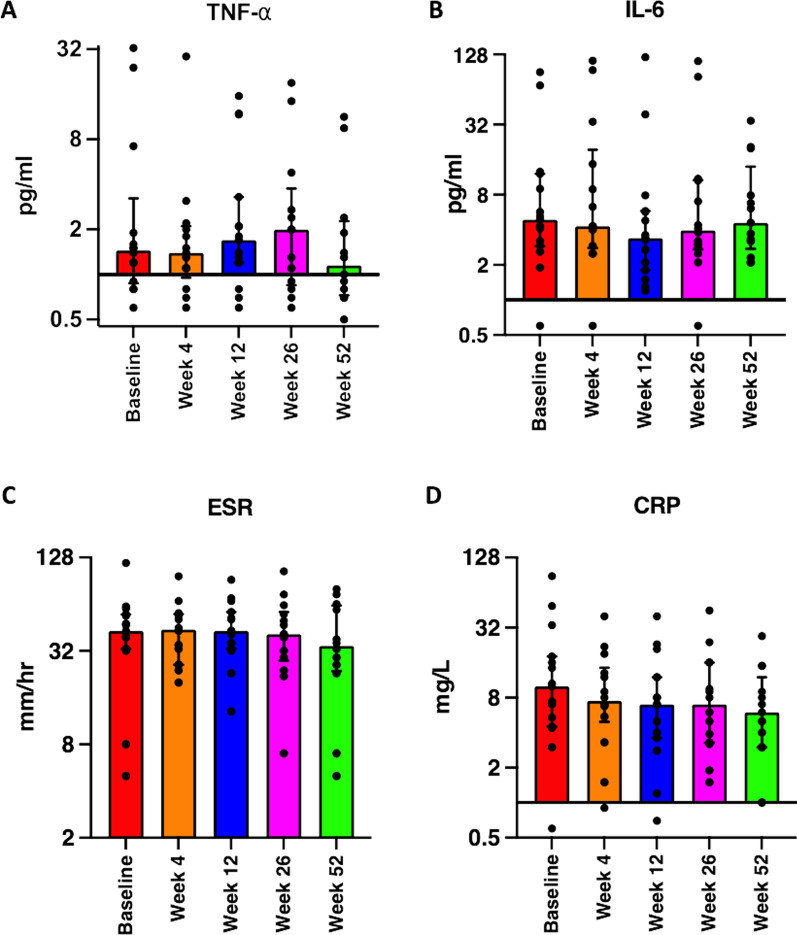
Inflammatory parameters. Levels of inflammatory cytokines, TNF-α or IL-6 did not show any significant changes at the follow up visits compared to baseline (**a**, **b**). No significant changes were seen in either ESR or CRP levels (**c**, **d**). Significance defined at *p* value ≤ 0.01 (Holm–Šídák correction for multiple comparisons); Wilcoxon-signed rank test

Both IL-6 and TNF-α levels from baseline to week 52 were not significantly changed (*p* = 0.743 for TNF-α and *p* = 0.714 for IL-6), median values: 1.15 (IQR 0.73–2.30) and 4.60 (IQR 2.75–13.9) pg/ml respectively (Fig. 3a, b, Table 2). No significant changes were seen in ESR (*p* = 0.775; Fig. 3c). Despite not being statistically significant, CRP levels did show a small effect when compared to baseline, with an ES of 0.37 at week 52 post-intervention (Fig. 3d, p = 0.183; significance determined at *p* < 0.01, Wilcoxon-signed rank test. For median and IQR values, see Table 2). Also, individual subject level summary of efficacy changes at week 52 vs baseline (swollen and tender joint counts and CRP levels) is presented in Additional file 2: Table S2.

Detailed summary of descriptive statistics and statistical significance for joint scores and inflammatory parameters at individual follow-up visits are given in Additional file 2: Table S4.

For all efficacy results, significance was determined at *p* < 0.01, Wilcoxon-signed rank test.

## Discussion

The current study is one among few to employ adipose-derived autologous MSCs [26] rather than more commonly used allogenic cells [29, 30], sourced from either bone-marrow or umbilical tissues [31, 32] to evaluate safety and efficacy in RA patients. To the best of our knowledge, this is one of the rare studies of MSCs in RA [33] to employ the ACR 66/68 swollen and tender joint count that also accounts for feet assessment in addition to the 28-joint count, included in more common composite indexes.

The results of this trial demonstrated that a single infusion of autologous HB-adMSCs is safe and efficacious in improving clinical symptoms of RA. Administration of HB-adMSCs resulted in a favorable safety profile, significant and persistent improvements in joint function, as well as modest improvement in inflammatory marker CRP, consistent with previous studies using umbilical cord-derived mesenchymal stem cells [20–22]. The current study population was not ostensibly limited to, but was largely composed of, standard treatment non-responders and required only that patient be on a stable drug regimen 4 weeks prior to enrollment (Additional file 2: Table S2).

Data from this study reflect strong and lasting declines (up to 52 weeks) in the ACR 66/68 swollen and tender joint scores. A vast majority of sufficiently powered studies to our knowledge have found declines in the more commonly used DAS28 index [20, 21, 23]. The current trial demonstrated clinically significant outcomes (with large effect sizes) in response to ACR 66/68 joint assessments, contrary to a previously reported placebo-controlled study, where improvements in joint scores and CRP were also implicated, but trends for early efficacy and permanence of improvements were much less obvious [33].

In response to inflammatory stimuli, live MSCs are capable of releasing an active secretome that consists of potent agents for immunomodulation, as opposed to dead or non-viable MSCs that fail to inhibit immune cell function [34]. MSC potency can be assessedlargely by cell viability, one of the most important cellular attributes influencing persistent therapeutic efficacy. Poor product viability can often lead to negative or null clinical outcome as was apparent in a placebo-controlled, randomized study with allogeneic MSCs, where the viability varied from 36 to 85% [35]. A single dose of fresh, viable cells employed in the current study resulted in significant improvements in joint function for up to 52 weeks. These cells met standardization requirements such as cell identity, purity, and most importantly, absolute cellular viability (> 94%) (Additional file 2: Table S1). There is no indication of an age effect to subject response suggesting that subjects of any age could benefit from the treatment.

MSCs exert direct effects through secretion of their own anti-inflammatory factors, and indirect effects by shifting core immune cells from proto anti-inflammatory states, thereby inducing the generation of CD8 + CD28- Treg cells while downregulating pro-inflammatory Th17 cells [36, 37]. Potential mechanism through which MSCs exert their therapeutic effects were implicated by previous studies, that included the role of IFN- γ and Treg cells. IFN- γ levels were initially found to predict subjects who responded to therapy, lending credence to ideas of a “licensing” process in MSCs via inflammatory mediators [21]. The same authors followed the trial with a preclinical experiment demonstrating the necessity of IFN-γ for MSC-induced recovery and ultimately a clinical trial where they demonstrated that MSC cells encoded to produce IFN- γ are therapeutically more potent than those that do not [29]. Other studies suggest a potential role for mediation of benefits by Treg cells, demonstrating a 2–3-fold increase in Treg cells 1 month post treatment with either allogeneic umbilically-derived MSCs [21] or autologous bone marrow-derived MSCs [32]. These findings have important mechanistic implications for future studies, as well as MSC sourcing and/or licensing.

Past studies have also reported significant declines in inflammatory parameters like CRP [20–23], ESR [21–23, 32], IL-6, TNF-α [20, 22] in addition to increases in Treg cells [21, 32] but results are less consistent than measures that include joint function. In the current study, however, the changes were only observed in CRP levels. Although not statistically significant, these changes were associated with a small effect size, indicating clinical relevance. Levels of other inflammatory factors, ESR, TNF-α and IL-6 remained significantly unchanged, during the entire course of this trial. It is important to note that concomitant therapy with IL-6 inhibitors, TNF-α inhibitors and glucocorticoids that may have obscured potential changes in these inflammatory markers. It is also possible that localized changes to joint condition vs systemic inflammation may result from different mechanisms of the MSCs occurring over different timescales. Also, substantially lower statistical power (*N* = 15) and low baseline values in some patients may partly explain this finding. All these implications reflect that to observe clinical efficacy in these inflammatory parameters, a study design with a larger population size, together with multi-dose administration, may be necessary.

Standard measures of hematologic, renal, and hepatic function were largely stable in the present study, except small changes in albumin to globulin ratio were observed (*p* = 0.047), despite no significance was seen in either albumin (*p* = 0.317) or globulin (*p* = 0.077) levels. Contrary to these results, some other studies reported increases in total protein [20], increases in albumin [20, 21], decreases in globulin [20, 22], along with functional improvements. More intriguingly, these changes were only seen in patients with clinical improvements; no significant result for any metric was observed in non-responders [21]. At least one study found no changes in albumin among several other metrics measured (globulin levels and albumin to globulin ratio were not reported) [23], consistent with the results in the current study.

Neither this study, nor other studies of MSCs in RA have found a suggestion of safety risks or significant AEs [19]. Limitations of this study include the small sample size, limited demographic distribution (sex and race) and lack of control group. Inclusion thresholds and baseline values of CRP and ESR were low relative to other studies and standards may have contributed to the lack of evidence of improvement in these parameters.

## Conclusion

The current study demonstrated that a single administration of fresh, autologous, HB-adMSCs is both safe and highly effective in ameliorating joint symptoms in patients with RA. Given the strong safety profile and clinically significant efficacy outcome for joint function, the results of this trial should be confirmed by a larger, randomized, placebo-controlled trial to have a better confirmation of therapeutic benefits of adMSCs in patients with RA.

### Supplementary information

**Additional file 2: Table S1**. MSCs quality control metrics for single infusion for N = 15 subjects (Table S1). Also, individual subject level summary of disease and treatment history and efficacy outcomes are given in Additional file 2: Table S2. Summary lab results for safety and efficacy measures at each measured timepoint (weeks 4, 12, 26 and 52) compared to the baseline are in Additional file 2: Tables S3 and S4.

**Additional file 1: Fig. S1.** Culture images at passage 4 for N = 15 subjects.

## Supplementary Information

**Additional file 1: Fig. S1**. Passage 4 culture images of each subject: Images were taken with a Leica inverted microscope at 50 × magnification. Consistent cell growth and morphology is observed across all donors. Color variation is due to varying flask wall thickness, angle, and light.

**Additional file 2: Table S1**. MSC quality control metrics for single infusion for N = 15 subjects. MSCs are expected to be positive for CD73 and CD29 and negative for CD45 and CD31. Columns 3–7 in the table indicate the percent of cells expressing the marker. **Table S2**. Individual subject level summary of disease & treatment history and efficacy outcomes. Both medications taken at the start of the trial and previously failed medications are described. Only medications with the capacity to alter the disease process transiently or permanently are reported (DMARDs or Glucocorticoids). Individual subject comparisons (baseline vs week 52) of Tender joint count, swollen joint count and C-reactive protein (CRP) that showed significant changes during the trial, are reported as indicators of disease activity. **Table S3**. Summary statistics of all safety measures. Summary evaluation of lab values at the baseline and each of the follow-up visits. Overall significance values are displayed *p < 0.05; Mixed Effect Analysis corrected with the Geisser-Greenhouse correction followed by Dunnett’s test for multiple comparisons. **Table S4**. Summary statistics of all efficacy measures. Summary evaluation of joint count data and inflammatory parameters at the baseline and each of the follow-up visits. ES are reported for statistically significant values (bold). *p < 0.01 and **p < 0.001 (Holm–Šídák correction for multiple comparisons); Wilcoxon-signed rank test.

## Data Availability

The data that support the findings of this study are available on request by emailing the corresponding author.

## Abbreviations

MSC: Mesenchymal stem cells
HB-adMSCs: Hope biosciences’ adipose-derived mesenchymal stem cells
adMSCs: Adipose-derived mesenchymal stem cells
ACR: American College of Rheumatology
AEs: Adverse events
DMARDs: Disease modifying anti-rheumatic drugs
TNF-α: Tumor necrosis factor-alpha
IL-6: Interleukin-6
CRP: C-reactive protein
ESR: Erythrocyte sedimentation rate
ES: Effect size
RA: Rheumatoid arthritis
SJC: Swollen joint count
TJC: Tender joint count
IQR: Inter-quartile range
