# Supplementary figures and images for: Safety and efficacy of autologous, adipose-derived mesenchymal stem cells in patients with rheumatoid arthritis: a phase I/IIa, open-label, non-randomized pilot trial
Source: Stem Cell Res Ther. 2022 Mar 3;13:88. doi: 10.1186/s13287-022-02763-w (PMC8896321; doi:10.1186/s13287-022-02763-w)

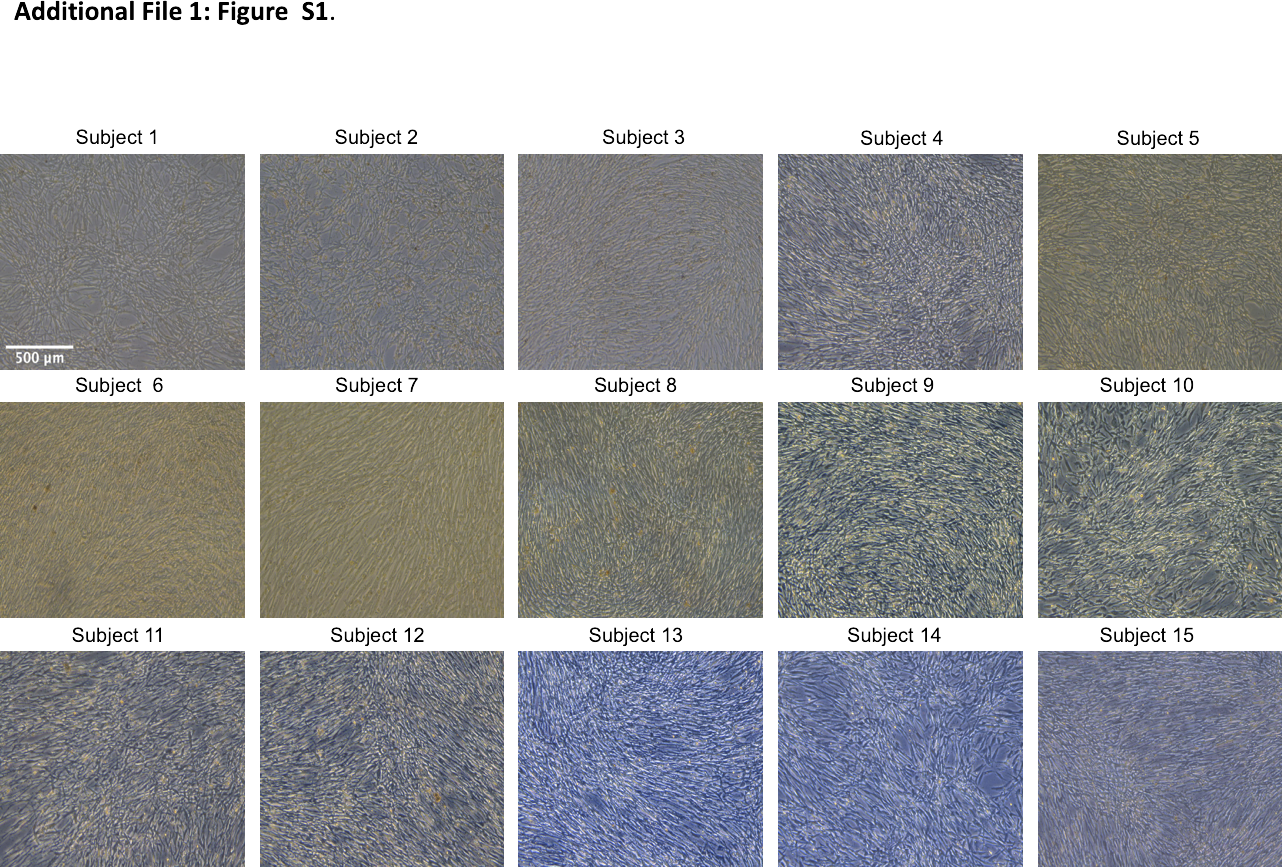

Supplement: Supplementary file 1 — Additional file 1: Fig. S1. Passage 4 culture images of each subject: Images were taken with a Leica inverted microscope at 50 × magnification. Consistent cell growth and morphology is observed across all donors. Color variation is due to varying flask wall thickness, angle, and light. [file 13287_2022_2763_MOESM1_ESM.tif]
